# An XROMM Study of Food Transport and Swallowing in Channel Catfish

**DOI:** 10.1093/iob/obaa018

**Published:** 2020-06-19

**Authors:** H I Weller, A M Olsen, A L Camp, A R Manafzadeh, L P Hernandez, E L Brainerd

**Affiliations:** 1 Department of Ecology and Evolutionary Biology, Brown University, Providence, RI, USA; 2 Department of Musculoskeletal Biology, University of Liverpool, Liverpool, UK; 3 Department of Biological Science, The George Washington University, Washington, DC, USA

## Abstract

Most predatory ray-finned fishes swallow their food whole, which can pose a significant challenge, given that prey items can be half as large as the predators themselves. How do fish transport captured food from the mouth to the stomach? Prior work indicates that, in general, fish use the pharyngeal jaws to manipulate food into the esophagus, where peristalsis is thought to take over. We used X-Ray Reconstruction of Moving Morphology to track prey transport in channel catfish (*Ictalurus punctatus*). By reconstructing the 3D motions of both the food and the catfish, we were able to track how the catfish move food through the head and into the stomach. Food enters the oral cavity at high velocities as a continuation of suction and stops in the approximate location of the branchial basket before moving in a much slower, more complex path toward the esophagus. This slow phase coincides with little motion in the head and no substantial mouth opening or hyoid depression. Once the prey is in the esophagus, however, its transport is surprisingly tightly correlated with gulping motions (hyoid depression, girdle retraction, hypaxial shortening, and mouth opening) of the head. Although the transport mechanism itself remains unknown, to our knowledge, this is the first description of synchrony between cranial expansion and esophageal transport in a fish. Our results provide direct evidence of prey transport within the esophagus and suggest that peristalsis may not be the sole mechanism of esophageal transport in catfish.

## Introduction

Capturing food is only the first challenge of a feeding event. For vertebrates, even once food enters the mouth, at a minimum, it must be transported back to the pharynx and enter the esophagus. Often, this also requires reorienting, manipulating, and processing a food item that could pose a number of problems, such as being irregularly shaped, covered in spines, or alive and struggling to escape. Terrestrial vertebrates generally rely on their muscular tongues for transporting and manipulating food back to the esophagus, and even aquatic tetrapods such as salamanders and turtles rely to some degree on lingual transport (Heiss et al. 2018). But the majority of aquatic vertebrates—ray-finned and cartilaginous fishes—lack muscular tongues for controlling the trajectory of a captured food item. Studies on terrestrial-feeding ray-finned fishes indicate that fishes use water to shunt food back to the esophagus (Michel et al. 2015; Heiss et al. 2018), but the general mechanisms for aquatic handling and swallowing in fishes are still unknown.

Although fish feeding has been the focus of decades of research in biomechanics, we know much more about how fishes capture and process food than about how they transport and swallow it (Gillis and Lauder 1995). This discrepancy exists partly for practical reasons: transport and swallowing take place inside a fish’s mouth, where researchers cannot directly observe or record them. But swallowing captured prey can present a major barrier for successful feeding; in extreme cases, predators unable to swallow large or irregularly shaped prey will die in the attempt (Robertson et al. 2019).

More generally, intraoral transport and swallowing place constraints on how, where, and on what fishes can feed. Piscivorous fishes typically strike their prey from the side, but must reorient captured prey in the mouth to swallow it head first (Hoyle and Keast 1987; Turesson et al. 2002; Cantanhêde et al. 2009; Mihalitsis and Bellwood 2017). Fish that feed on land use a hydrodynamic tongue to transport food to the esophagus (Michel et al. 2015) or must return to the water to swallow (Cucherousset et al. 2012). Many predatory fishes use their pharyngeal jaws to transport large prey, probably by dragging it into the esophagus (Lauder 1983; Liem 1970). To swallow particulate foods, some cyprinids rely on a combination of crossflow filtration and palatal protrusions (Callan and Sanderson 2003). The fused lower pharyngeal jaws of cichlids, which might have contributed to their ecological success (Liem 1973), also prevent them from swallowing large prey whole (McGee et al. 2015; Burress 2016). Understanding the challenges of swallowing captured food, and how those challenges have shaped feeding morphologies and behaviors, requires understanding how fish transport and swallow food under less extreme conditions.

Part of the challenge in studying a process that cannot be directly observed is that it can be difficult to form testable hypotheses without being able to directly observe those processes. X-ray Reconstruction of Moving Morphology (XROMM) provides one solution to this problem by allowing us to track the 3D kinematics of individual bones in a fish’s head during a feeding event and simultaneously reconstruct how captured food moves through the fish’s mouth (Brainerd et al. 2010). Van Meer et al. (2019), in their study of food transport and swallowing in white-spotted bamboo sharks (*Chiloscyllium plagiosum*), used XROMM data to document a stepwise mode of transport: sharks transported food in punctuated rostrocaudal bursts of motion, and swallowed it at relatively high velocity, which they attributed to alternating cycles of hydrodynamic transport with the pharyngeal arches holding the food stationary.

The Van Meer et al. (2019) study illustrated both how XROMM feeding data can be used to understand intraoral transport and how poorly we understand food transport and swallowing in aquatic vertebrates. The stepwise transport and swallowing described by Van Meer et al. (2019) are unlike any known mode of swallowing in terrestrial vertebrates (Levine et al. 2004; German et al. 2009), and support the hypothesis that aquatic and terrestrial feeding place fundamentally different constraints on vertebrate feeding mechanisms (Liem 1990; Heiss et al. 2018). But while sharks and ray-finned fishes both feed underwater, their buccal anatomies are substantially different—most obviously, while sharks have at least five gill slits and little branchial dentition, ray-finned fishes have only one opercular opening, and pharyngeal jaws just rostral to the esophageal sphincter. These differences should be especially relevant during deglutition. Pharyngeal jaws offer an additional point of control in the caudal part of the pharynx, while a single opercular opening compared to multiple gill slits could substantially alter the mechanisms of hydrodynamic manipulation.

To study food handling and swallowing in a ray-finned fish, we used an XROMM dataset collected by Olsen et al. (2019) to study intracranial coordination during feeding in channel catfish (*Ictalurus punctatus*). The authors found that while coordination was high during capture, it dropped considerably during transport, a change they attributed to the need for the fish to generate irregular hydrodynamic flows in response to the location and trajectory of the food. That study focused on the coordination of intracranial motions during feeding, rather than on the motion of the food itself. The dataset also includes marked food items, however, allowing us to reconstruct their 3D trajectories through the buccal cavity and into the esophagus, so we use it here to address the related problem of how the food moves through the mouth, and how intracranial motions might drive that transport. Although this dataset was not originally collected for investigating handling and swallowing mechanisms, we were able to address two sets of predictions using this dataset.

First, because many previous studies have demonstrated the importance of the pharyngeal jaws for manipulating, processing, and swallowing food across ray-finned fishes, (Liem 1970; Wainwright 2005; Mehta and Wainwright 2008; Gidmark et al. 2014), we expected food handling to mostly take place in the region of the pharyngeal jaws.

Second, because Olsen et al. (2019) found that intracranial coordination was higher during capture than transport, we expected the correlation of food and intracranial motions to be highest during capture and lowest during esophageal transport, when peristalsis is expected to drive the motion of the food (Schwenk and Rubega 2005).

Outside of these predictions, because we know so little about transport and swallowing in ray-finned fishes, a broader goal of this project is to describe patterns in how food is transported through the buccal cavity and esophagus. We aim to use the descriptions we report here to determine specific testable questions about handling and swallowing in ray-finned fishes to be addressed in future work.

## Materials and methods

### Data collection and tracking

**Fig. 1:**
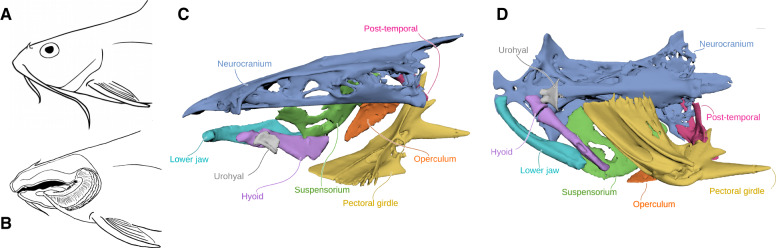
*Ictalurus punctatus* cranial anatomy. (**A**) Diagrams of external cranial anatomy and (**B**) the buccal cavity made from a dissected museum specimen from the University of Michigan (UMMZ 236738-A). (**C**) Medial and (**D**) ventral views of the left-side cranial elements (horizontally flipped for orientation to graph axes in other figures) that we marked with radiopaque beads, segmented from a computed tomography scan.

We use the same XROMM dataset in this study as Olsen et al. (2019), which provides details on animal care, marker implantation, X-ray filming and tracking, XROMM animation, and the determination of best-fit axes of intracranial motion. Briefly, to generate the dataset, implantation surgeries were performed on channel catfish (*I. punctatus*, 32.8 ± 2.4 cm SL) to implant small, radiopaque, spherical tantalum markers (0.5 and 0.8 mm in diameter). A minimum of three markers was implanted on the left side into six skeletal elements (neurocranium, suspensorium, operculum, lower jaw, hyoid, and pectoral girdle) and one marker into the urohyal (Fig. 1). Markers along the midline of the hypaxial muscles were injected using hypodermic needles.

Fish were filmed using X-ray videos from two views (biplanar fluoroscopy) at 300 frames $s^{-1}$. Three different types of food were offered: pieces of earthworms (earthworms cut to be about 2.5 cm long), pieces of squid, and pellets. Because the original objective of this dataset was to elicit maximal intraoral pressure changes and not to study the effect of food types on handling, the different food items were not offered to individuals systematically (Table 1). A single 0.8 mm tantalum marker was implanted in each offered food item, allowing us to track translation of the food throughout each trial. Markers were tracked using XMALab (Knörlein et al. 2016). Video data were stored with their essential metadata in accordance with best practices for video data management in organismal biology (Brainerd et al. 2017).

**Table 1:** Feeding trial metadata by individual and food type

		Food		
Individual	Trials	Pellet	Squid piece	Worm piece
Cat 01	11 (6)	8 (5)	1 (0)	2 (1)
Cat 02	11 (7)	2 (2)	0 (0)	9 (5)
Cat 05	3 (3)	0 (0)	0 (0)	3 (3)
Total	25 (16)	10 (7)	1 (0)	14 (9)

The number of trials that include swallowing and esophageal transport is given in parentheses for each cell.

### XROMM animation workflow

Olsen et al. (2019) produced rigid body transforms describing the motion for each marked cranial element using the unifyMotion function from the matools package in R (Olsen 2019) to find rigid body motion relative to the neurocranium in each trial, because this bone provided a consistent frame of reference for buccal cavity transport. For this project, we imported the rigid body transforms into Autodesk Maya 2018 to create 3D animations for each trial, also with respect to the neurocranium (Baier 2018). We represented the motion of the food with a particle emitter. We treated the rostral-most tip of the neurocranium, on the midline, as the origin of this coordinate system.

We determined the location of the esophageal sphincter using air contrast in post-mortem computed tomography scans of each fish. We used a combination of soft polyurethane foam and paper towels to fill the oral and buccal cavities and esophageal sphincter of each individual before scanning. The low densities of these materials created contrast between the expanded cavities and their surrounding soft tissues, allowing us to identify the location of the esophagus relative to the bones we marked. In Horos (Nimble Co. LLC, Annapolis, MD, USA, https://horosproject.org/), we placed point markers in a perimeter around the visible border of the esophageal sphincter (determined from air/soft tissue contrast) and fit a best fit plane to the points, then measured the distance from that plane to the rostral tip of the neurocranium. Because the neurocranium was marked in all of our trials, we used this distance as a proxy for the location of the esophageal sphincter on a midsagittal rostral–caudal axis, allowing us to calculate the distance of the food from the esophagus over a trial.

### Food trajectory analyses

By marking each food item with a single tantalum marker, we tracked the translation of the food throughout each trial. We calculated the 3D trajectories of the food with respect to the anatomical planes of the neurocranium (rostrocaudal, mediolateral, and dorsoventral).

For each trial, we also scaled all values to the head lengths of the individual catfish (rather than millimeters). We also measured motion in the rostrocaudal direction to relative distance from the oral jaws, where food at the oral jaws would have a rostrocaudal value of 0 and food at the esophagus would have a value of 1.

To look for shifts in the motion of the food after suction feeding, we used the Fstats function from the strucchange package in R (Zeileis 2006) to find breakpoints in the velocity of the food in the rostrocaudal direction and the overall instantaneous speed of the food by taking the discrete derivative of rostrocaudal or 3D motion, respectively. If the analysis recovered a breakpoint with strong support ($P<10^{-3}$, half of a Bonferroni-corrected *P*-value threshold from an initial *P* of 0.05 given the number of trials we tested), we used the index of that breakpoint to divide food transport into phases. We also divided motion of the food before and after passing through the best-fit esophageal plane, resulting in three phases (before the breakpoint, between the breakpoint and the esophageal sphincter, and in the esophagus).

For each phase, we calculated the average speed of the food by finding the average of that phase for each trial and pooling the averages to avoid weighting biases. We also calculated the path complexity, a ratio of the total distance traveled to the displacement, in the same way.

**Fig. 2:**
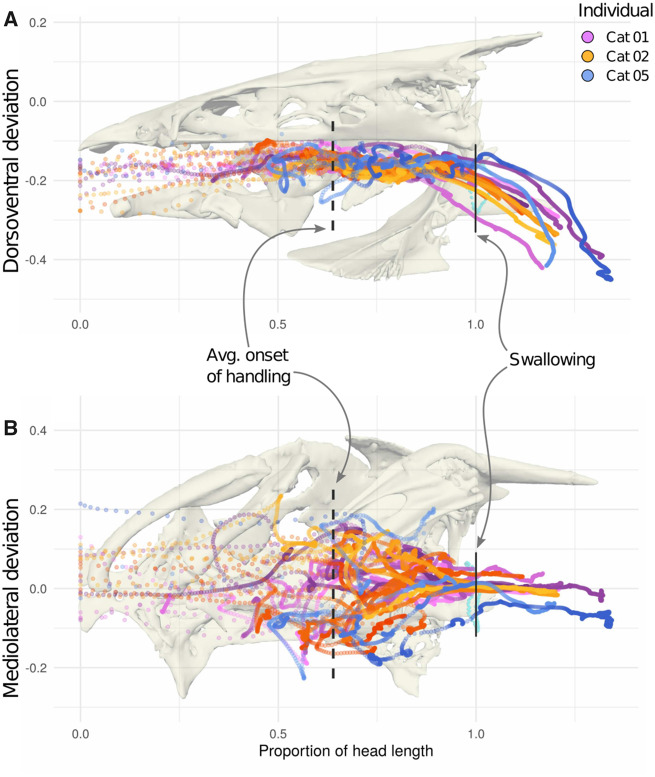
Food trajectories from all trials in medial (**A**) and ventral (**B**) views, colored by individual (shades of purple = Cat 01, orange = Cat 02, and blue = Cat 05). Dashed lines indicate the average location of the breakpoint (transition from capture to handling); solid lines indicate the location of the esophagus (onset of swallowing). All values have been normalized to the head length of the individual fish. The wide dorsoventral spread of the trajectories in the esophagus is an artifact of fixing motion with respect to the neurocranium. The bone models in the background of each graph are from a pre-strike position of Cat 01 and are accurately scaled to the graph axes.

In total, we analyzed 25 feeding trials across three individuals (Fig. 2) in which the food at least reaches the esophageal sphincter (3–11 trials per individual); 16 of these trials included the motion of the food past the sphincter and through the esophagus itself (Table 1). Although we report the different food types, our resulting dataset is not balanced enough to statistically distinguish the effect of individuals from the effect of the food type on the food trajectories. We divided food transport and swallowing into three phases, with transition points based on (1) quantitative changes in the food’s trajectory and (2) the point at which food passed through the esophageal sphincter. We refer to these phases as capture, handling, and swallowing and esophageal transport.

### Cross-correlations of food transport and intracranial motion

To test for the correlation of food and intracranial motions, we tested for cross-correlations between the rostrocaudal velocity of the food and the velocity of the rotation of each of the axes of motion calculated in Olsen et al. (2019) across each phase of transport, allowing for lag. We used the ccf function from the stats package in R (R Core Team 2019). Briefly, this method tests the similarity of two series by “sliding” (lagging) one series across the other and finding the lag at which the two series—in this case, food transport and intracranial motion—are most similar.

To constrain the correlations across trials to have the same lag (i.e., the same relationship between food transport and intracranial motion), we first concatenated motion across all trials, with missing values (NA in R) as spacers between individual trials so that lagging did not cause values from one trial to overlap with the next.

## Results

### Food trajectory differences across ingestion phases

#### Capture

**Fig. 3:**
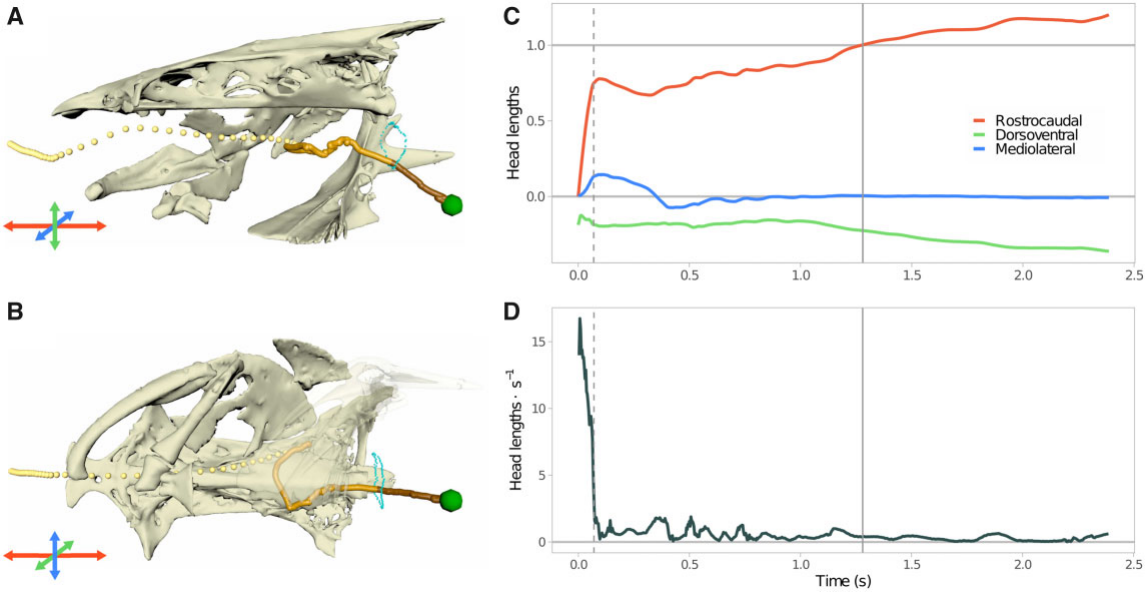
Food transport from a single representative trial. Final frames from an animated feeding trial from medial (**A**) and ventral (**B**) views. Insets show the rostrocaudal (red), dorsoventral (green) and mediolateral (blue) anatomical axes used for tracking food motion relative to the neurocranium. The path of the food (green sphere) is traced with spherical gold particles, with different shades for each phase (lightest = capture, mid = handling, darkest = swallowing and esophageal transport). Cyan spheres indicate the points around the esophagus from the air-contrast computed tomography scan (see “Materials and methods” section). (**C**) Location of the food in the head over time in units of head length (0 = mouth, 1 = esophagus). (**D**) Speed of food over time, in head lengths per second. Dashed and solid lines indicate handling and swallowing, as in Fig. 2.

Food first entered the buccal cavity as a continuation of the suction feeding event the fish used to capture it. Its trajectory through the rostral part of the buccal cavity was predominantly caudal (Fig. 3 and Supplementary Video S1), with little lateral or dorsoventral deviation, and a path complexity (a ratio of the total distance traveled to the net progress toward the esophagus) close to 1 (Table 2). Food typically entered the mouth at peak velocity (17 ± 9 head lengths s^−1^, or about 1.2 ± 0.6 m × s^−1^), decelerating as it traveled through the rostral part of the buccal cavity.

**Table 2:** Average food trajectory parameters during the two buccal cavity transport phases

	Capture	Handling	Fold change
Time (ms)	89 (± 53)	1397 (± 541)	**15.7**
Speed (HL $s^{-1}$)	10.93 (± 7.37)	0.82 (± 0.45)	**0.07**
Path complexity	1.13 (± 0.23)	3.25 (± 2.68)	**2.88**

Fold change (in bold text) is the ratio of handling to suction for a given parameter; for example, food spends about 16 times longer in handling than capture.

During this phase, food usually traveled more than half of the distance to the esophagus in less than a 10th of a second. Before handling began (see below), food was transported 60.5 ± 15.5% of the distance to the esophagus in an average of 89 ± 53 ms.

#### Handling

**Fig. 4:**
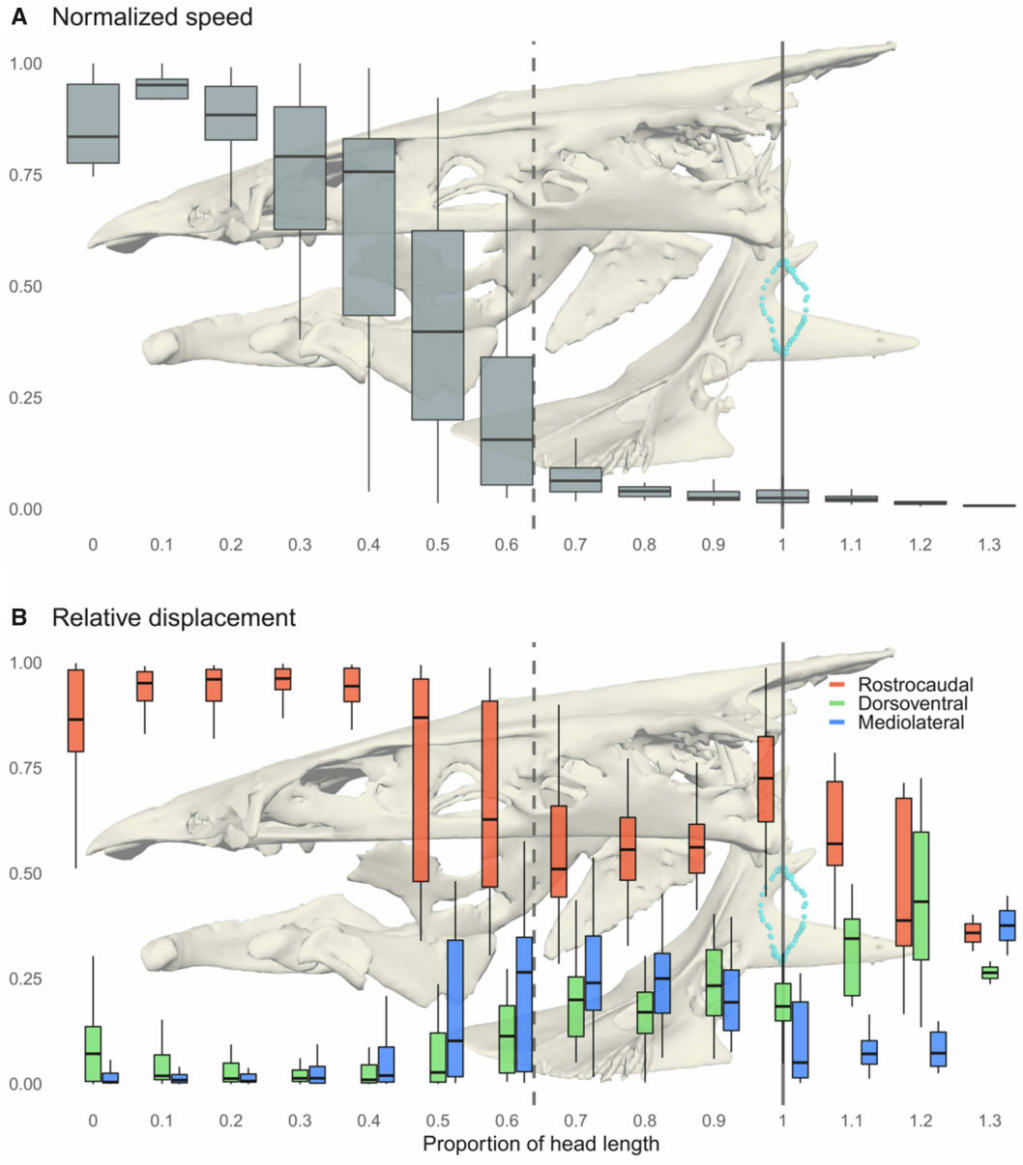
Average food speed and motion along each body axis. (A) Speed of food items across 10% increments of the head, scaled to the maximum speed for each trial. (B) The proportion of motion along each body axis, again in 10% increments (note that these are proportions of the total motion for a single increment; there is much more total displacement during capture than other phases). Dashed and solid lines indicate average breakpoints and the location of the esophagus, as in Fig. 2. Background bone models are to scale on the *x*-axis.

We recovered strongly supported breakpoints for all trials ($P\ll 10^{-3}$), corresponding in each case to the food coming to a near or full stop in the oral cavity. Handling started when the food shifted from the high, rapidly decelerating speed of the initial suction event to near-zero speeds punctuated by irregular bursts of motion (Fig. 4A). Average path complexity also increased from just over 1 (primarily caudal motion) to 3.25 (Table 2, Fig. 4B), meaning on average food traveled more than three times the remaining distance to the esophageal sphincter before reaching it.

Average food speed during handling was much lower and more variable than during suction: 0.82 ± 0.45 head lengths s^−1^. Consequently, transport took much longer during this phase than during suction, taking an average of 16 times longer to travel the caudal 40% of the distance to the esophagus as it took to travel the rostral 60%, with little deviation across trials.

#### Swallowing and esophageal transport

Of the 25 recorded trials, 16 included partial food transport in the esophagus. Food moved slowly, with comparable speed to the handling phase (0.25 ± 0.19 HL $s^{-1}$), although its motion was smoother (Fig. 2). We observed little variation in the trajectories of food items once they were in the esophagus.

### Breakpoint locations

**Fig. 5:**
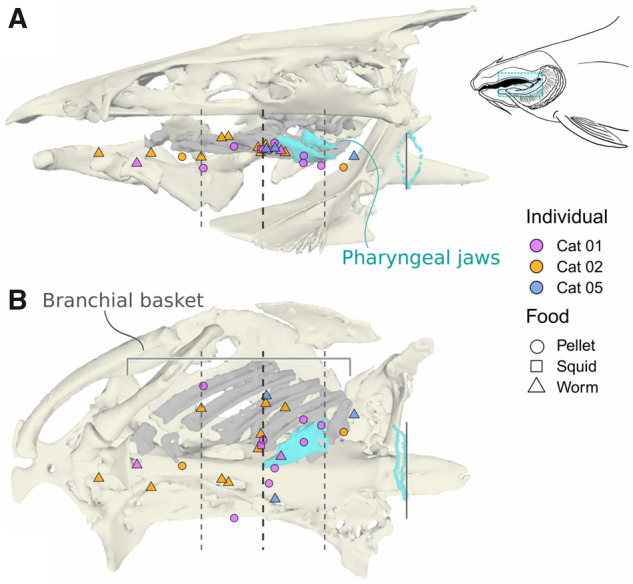
Locations of breakpoints from medial (**A**) and ventral (**B**) views. In addition to the marked cranial elements, the background image also includes the left half of the closed branchial basket (gray) and the left upper and lower pharyngeal jaws (cyan) from the same computed tomography scan for reference. The dashed lines indicate the average and standard deviation of the breakpoint locations; solid lines indicate the esophagus. Inset diagram of the internal anatomy includes a bounding box indicating the range of breakpoints, which approximately bracket the branchial basket. Food types are indicated, but the dataset is not balanced enough to test for the effect of different food types (see text).

We tested whether the locations of the breakpoints (Fig. 5) were correlated with either the individual catfish or the type of food using multiple linear regression, fitting both individual and food type as fixed-effect categorical variables (although individual would be considered a random effect, we treated it as a fixed effect because there were fewer than five categories, per Harrison et al. 2018). Neither variable was significantly correlated with the location of the breakpoints.

### Correlations between food transport and cranial motion

**Fig. 6:**
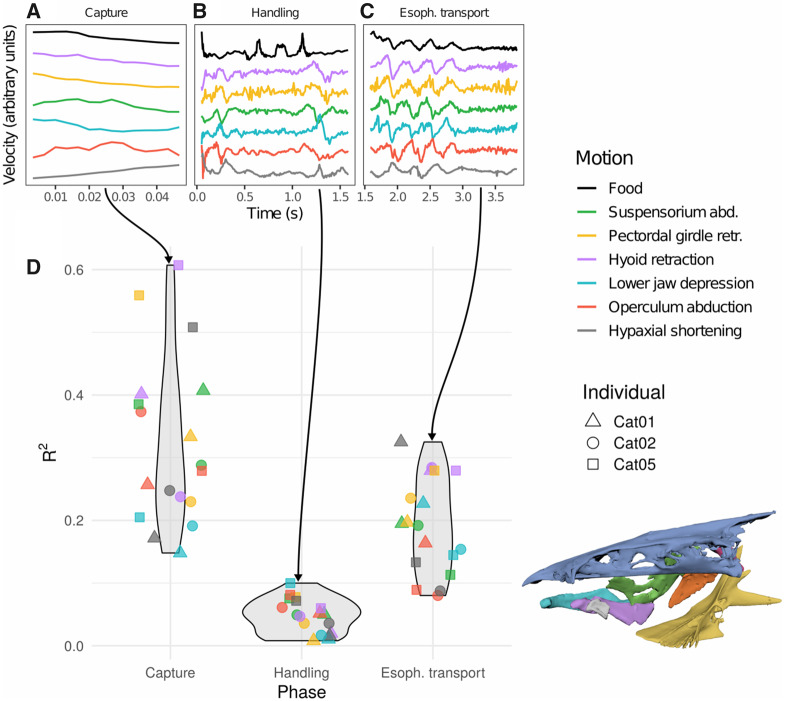
Cross correlations between cranial motions and food motion across the three phases of a feeding event. Axes of motion are the same as those described in Olsen et al. (2019). A-C: food and cranial motion velocities from a single trial of Cat 02, plotted by phase. Velocities were scaled by phase. D: cross-correlation coefficients between cranial motion and food motion by individual, with a color-coded skull for reference.

We found the highest correlations (*R*^2^) between food and cranial motion during capture (Fig. 6A), with the strongest correlations between food motion and hyoid depression/retraction, pectoral girdle retraction, and hypaxial shortening, which account for the bulk of the pattern (Fig. 6).

Food had the weakest correlations with cranial motion during handling across all individuals; transport in this phase was not well explained by motion of the six bones we tracked. There was also little cranial motion during handling compared to suction.

By contrast, correlations were not only higher during esophageal transport than handling, but some correlations were as high as the correlations we observed during food capture. Specifically, hyoid retraction and hypaxial shortening both had $R^{2}>0.3$, while no correlation coefficients during handling exceeded $R^{2}\approx 0.1$. Once food entered the esophagus, catfish performed repeated cranial expansions, resembling heavy ventilation (Hughes 1960). These instances of head expansion always corresponded to a near-simultaneous jump in the speed of the food moving down the esophagus (lag of about 25 ms; see Table 3 and Supplementary Information).

**Table 3:** *R* ^2^ and lag time for cross-correlations of food and hyoid retraction velocities

	Individual					
	Cat 01		Cat 02		Cat 05	
Phase	*R* ^2^	Lag (ms)	*R* ^2^	Lag (ms)	*R* ^2^	Lag (ms)
Capture	0.4	0	0.24	0	0.61	−3
Handling	0.02	−13	0.05	0	0.06	−20
Swallowing	0.28	27	0.28	23	0.28	27

The lags given are the lag of food velocity with respect to hyoid retraction velocity. Lags and correlations for other motions are available as Supplementary Material.

## Discussion

Food trajectories in this study showed remarkably consistent patterns across individuals. Although the food had a unique trajectory in each trial, it always started by moving caudally at high velocity, coming to an abrupt stop midway through the buccal cavity, then moving erratically at a much lower velocity the rest of the way to the esophagus (Figs. 2 and 4A).

Despite the consistent appearance of a breakpoint between food capture and handling, the locations of the breakpoints indicate that the pharyngeal jaws are important for manipulating food in the buccal cavity toward the esophagus, but not solely responsible for it. And while the high correlations (Fig. 6A) between food transport and cranial motion we measured during capture are almost certainly a continuation of the suction feeding strike that brought the food into the mouth, the high correlations (Fig. 6C) between food transport and both pectoral girdle retraction and hypaxial shortening once the food is in the esophagus are more difficult to explain. Once fish have swallowed food, we would expect peristalsis alone, and not skeletal motion, to be driving transport to the stomach. These results allowed us to test hypotheses about the roles of the pharyngeal jaws and of peristalsis in how fish handle and swallow food, and yielded observations that should inform future work in this area.

### Evidence for food handling by both the pharyngeal jaws and the branchial basket

In each of our 25 trials, the food item came to an unambiguous stop midway through the buccal cavity, then moved erratically toward the entrance to the esophagus with substantial mediolateral displacement (Fig. 4).

We hypothesized that handling would mostly take place in the region of the pharyngeal jaws. This would support a substantial amount of work showing the pharyngeal jaws as important in food handling, even in fishes without highly derived pharyngeal jaw modifications (Liem 1970; Lauder 1983; Claes and De Vree 1991; Vandewalle et al. 1994; Gillis and Lauder 1995; Wainwright 2005; Mehta and Wainwright 2008). The switch from a straight, high-velocity trajectory to a slow and winding one is certainly in line with what we would expect based on this previous work, in that the pharyngeal jaws are the most obvious point of control for the catfish to manipulate food into the esophagus. If the pharyngeal jaws alone were responsible for food handling, however, we would expect that all of the handling breakpoints would be at or caudal to the pharyngeal jaws.

But many of our detected breakpoints are rostral to the pharyngeal jaws, and the support for these breakpoints is just as unambiguous as the more caudal ones. One explanation for this observation is that because we are using point markers as proxies for food location, these breakpoints may not indicate the exact position of the food. However, these breakpoints span a wider rostral-caudal range than could be explained by this source of uncertainty. For example, the worm pieces, our most irregularly shaped food type, were about 25 mm (1 inch) long, or 35% the head length for each fish, while breakpoints for worm feeding trials span >70% of head length (Fig. 5), making these locations useful at least as a coarse indicator of handling. Instead, the distribution of trajectory breakpoints supports direct contact between the branchial basket and the food item as driving this second phase of transport: the locations of the breakpoints span the length of the branchial basket, and on average fall at the rostral point of the lower pharyngeal jaws (Fig. 5).

At least in *Ictalurus*, more rostral branchial arches are probably readily recruited for handling food much as they are in bamboo sharks (Van Meer et al. 2019). Admittedly, the complex musculature of the pharyngeal jaws in ray-finned fishes probably allows for finer control of a food item than do arches 1–4 (Springer and Johnson 2004). But *Ictalurus* does not have specialized pharyngeal jaws: they are relatively thin, unfused, and covered in small (generally <1 mm) conical teeth that likely serve more to increase friction than to process prey (Sis et al. 1979; Mihalitsis and Bellwood 2019). Unlike fishes with pharyngeal jaws specialized for prey processing, those of *Ictalurus* may provide only the most caudal point of control that the fish have over the orientation and position of the food before it enters the esophagus. As long as the fish can appropriately orient the food at some point before swallowing it, the pharyngeal jaws provide no special handling advantage over the other mobile buccal cavity elements; knifefishes (Notopteridae), for example, rely on raking motions with the hyoid apparatus for the bulk of prey transport and processing (Sanford 2001). In ray-finned fishes, branchial arches 1–4 are also important elements for food transport, ones whose function may have been overshadowed by that of the pharyngeal jaws (Vandewalle et al. 1994).

### Swallowing and the role of the pharyngeal jaws

Despite being able to observe how food moved as it passed into the esophagus with high detail and precision, we noted no change in the trajectories or velocities of food items as they were swallowed. Velocity remained uniformly low, and food items moved on into the esophagus with little or no retrograde motion. By contrast, in bamboo sharks, food jumped in velocity when it passed into the esophagus, sometimes with substantial retrograde motion (see Van Meer et al. 2019, especially Figs. 2, 3, and 5). They interpreted this burst of velocity as hydrodynamic: to swallow a food item, sharks shunted water through the oropharyngeal cavity, forcing the food caudally.

In our catfish data, the absence of any change to the pattern of food motion suggests a different mechanism of transport into the esophagus. Although swallowing in fishes is still poorly understood, electromyography work (Lauder 1983, 1985) supports the pharyngeal jaws as the main drivers of swallowing, by dragging and pushing food into the esophagus (Liem 1970). We did not mark the pharyngeal jaws in our XROMM dataset, both because they were difficult to access and because this was not the original intended purpose of the dataset, but in several of our trials, the pharyngeal jaws are visible on the X-ray video translating and rotating considerably just as food is moved into the esophagus (Supplementary Video S2).

### Cross-correlations of food and intracranial motion

#### Capture

The first phase of food transport we describe was essentially the last phase of a suction feeding event. Food entered the mouth at high speed due to suction, and continued to move along a linear trajectory 40–60% of the way into the buccal cavity. Consequently, we expected and have a comparatively good conceptual basis for the consistency we observed in this phase across trials (Fig. 6).

Although we found relatively high correlations between intracranial motion and food motion in this phase (Table 3 and Supplementary Material), we attribute them to a common source—the initiation of a suction feeding event—rather than to intracranial motions driving food transport in this phase. Instead, transport through the rostral 40–60% of the buccal cavity is probably due to inertia more than simultaneous motion. We stress, however, that because we were unable to track the motion of the water in the buccal cavity, this dataset does not allow us to explicitly test hypotheses about hydrodynamic food manipulation.

#### Handling

Handling exhibited by far the lowest correlations between food and the bones we marked and tracked. This is not necessarily surprising, given the above discussion of the likely recruitment of the branchial basket and pharyngeal jaws for food manipulation during this phase. We did not have either of these elements marked, and if they are driving food transport through the caudal section of the buccal cavity, then we would expect to see low correlations between the food and the elements we did mark.

That said, even if these cranial elements were driving food motion during the handling phase, we might still recover low correlations because of the variability in the food trajectories. The endpoint of the handling phase is the manipulation of food from the buccal cavity into the esophagus. The motions required to bring food into the esophagus will depend on where exactly the food is during the onset of handling—both how rostral it is, and the degree of mediolateral deviation off the midline. If, for example, a different set of motions is needed to bring food caudally than medially, and fish in different trials are combining those motions in variable ways, then a statistic designed to test for a consistent relationship between motions will not recover the underlying dynamics.

#### Esophageal transport

While the kinematics of the capture, handling, and swallowing phases of food transport we describe here all support prior work in fish feeding, the tight correlation between food and bone motion once food entered the esophagus is a novel kinematic pattern that is harder to explain. To our knowledge, although vertebrates employ a wide variety of methods for swallowing food, no mechanism besides peristalsis has ever been described for transporting food once it has entered the esophagus (Schwenk and Rubega 2005). It would be surprising if peristalsis played no role whatsoever here in esophageal transport: the channel catfish has bands of circular and longitudinal skeletal muscle that should be capable of the contractile waves that drive peristalsis (Sis et al. 1979).

Still, the tight correlation between esophageal transport and cranio-pectoral motion across all of our trials is hard to dismiss as pure coincidence. Of 16 trials that contained motion of the food in the esophagus (Supplementary Fig. S1), the 10 longest trials include at least one cycle of head expansion characterized by food motion lagging pectoral girdle and hyoid retraction by only about 25 ms (Fig. 6C illustrates the longest of these trials). Contrast this with the handling phase, in which both the head and the food exhibited similar magnitudes of motion, but in which we found essentially no support for cranial motion driving food transport (Table 3, compare second and third rows). Coincidence would be a reasonable explanation if food moved during cranial expansion only occasionally; as a satisfying explanation for the consistent relationship between food motion and cranial expansion across trials and individuals, it is harder to stomach.

Instead, we posit several other possible explanations for this observation and discuss what kinds of evidence would help distinguish which of them (if any) are correct. In order from least to most likely, we suggest this correlation could be a result of (1) swallowing water in order to push food through the esophagus, (2) peristalsis somehow aided by pectoral girdle retraction, or (3) heavy gill ventilation.

##### Using water to push food through the esophagus

One of the simplest explanations for this correlation is that, somewhat like bamboo sharks, catfish in our dataset used an influx of water taken into the mouth during cranial expansion to push food through the esophagus. If the water entered the esophagus after the food, it could transport the food caudally either through hydrodynamic forces (if the food was small relative to esophageal diameter) or through a buildup of pressure rostral to the food.

Teleosts have an esophageal sphincter that could prevent them from passively swallowing water (Stevens and Hume 2004), and some species swallow water intentionally. Mudskippers use mouthfuls of water to swallow food on land (Michel et al. 2015), pufferfish famously pump water into their stomachs to inflate themselves (Brainerd 1994; Wainwright et al. 1995), and at least one predatory catfish (the ogre catfish, *Asterophysus batrachus*) has also been documented to swallow huge volumes of water (Zuanon and Sazima 2005).

This hypothesis, however, does not explain our observation that food motion and cranial expansion are nearly simultaneous, instead of staggered. If food was being transported by water, we would expect a staggered sequence of events: (1) mouth opening and cranial expansion, pulling water into the buccal cavity, (2) mouth closing and cranial compression, forcing water out through the operculum and esophagus, and (3) caudal food motion, as the shunted water reaches the food in the esophagus. Instead, the pectoral girdle and hyoid were still retracted when the food moved caudally during these bursts.

##### Peristalsis aided by pectoral girdle retraction (“shrug-swallowing”)

Peristalsis could be driving the bulk of food transport, while the retraction of the pectoral girdle aids this process for small or unusually shaped food items by changing the dimensions of the esophagus itself. The esophageal sphincter is immediately caudal to the pharyngeal jaws, approximately at the level of the pectoral girdle. Retraction of the pectoral girdle could, through soft tissue compression, widen and shorten the esophagus, decreasing the distance between the food and the stomach.

It is noteworthy that in addition to hitting peak velocity during cranial expansion, food in the esophagus frequently slowed or stopped moving in between cycles of cranial expansion and compression. Because we were feeding the catfish relatively small food items (pellets and pieces of earthworms) in order to elicit as many feeding strikes as possible, the contractions of peristalsis alone may have been insufficient to transport the food, given that channel catfish typically forage larger food items. In this case, the “shrugging” of the pectoral girdle may have been recruited for esophageal transport where it would typically be unnecessary.

#### Gill ventilation

Fish often engage in what appears to be several cycles of heavy gill ventilation after swallowing food (Liem 1970; H. I. Weller, personal observation). The results we present here suggest that that “ventilation” may contribute to the last stage of a feeding event, serving primarily to bring food into the stomach, but it could also simply be heavy ventilation in the wake of an aerobically costly feeding behavior, which happens to move the food. If pectoral girdle retraction alters the shape of the esophagus, the motion of the food through the esophagus could be more of a byproduct than a driver of the kinematics.

On reviewing other XROMM fish feeding datasets (e.g., largemouth bass in Camp and Brainerd 2015, black carp in Gidmark et al. 2013), we could not find other instances of cranial expansion during esophageal transport, although relatively few of the trials recorded sufficiently long sequences to capture esophageal transport, and they used different prey types (live goldfish and ceramic cylinders filled with food, respectively). Still, the absence of a clear signal for cranial motion driving transport in other fishes at least confirms that cranial expansion is not a requirement for moving food through the esophagus, supporting peristalsis as the primary mode of transport. Instead, this may be a specific mechanism of Siluriformes for swallowing large, irregularly shaped prey while feeding on the benthos.

### Further potential experiments

Much of the ambiguity in understanding our esophageal transport kinematics stems from our inability to visualize the deformation of soft tissue in the X-ray videos. We cannot see when the esophageal sphincter is opened or closed to control water flow, when the esophagus itself is undergoing peristaltic contractions, or whether pectoral girdle retraction affects the shape of the esophagus.

The most direct test for any of our above hypotheses would be to mark the esophagus, stomach, and esophageal sphincter in multiple places with radiopaque markers in order to measure how these structures change shape during cycles of cranial expansion, both during ventilation and esophageal transport, and to track the flow of water with radiopaque fluid. This would allow us to cross-correlate the shape changes of these digestive structures with the kinematics of the head.

Recording feeding events using prey of different sizes and shapes would also be informative. Small, symmetrical pellets probably present a very different set of feeding challenges than swallowing, for example, birds (Cucherousset et al. 2012) or prey fish as long as the predators themselves (Liem 1970; Zuanon and Sazima 2005). More accurate marking (such as including multiple markers at fixed locations in the food) would also help us better track how food is manipulated and rotated, rather than just translated, after capture.

## Conclusions

Most of our results support prior work in fish feeding biomechanics: we found that food is passed into the esophagus after a substantial period of handling and manipulation, and that the pharyngeal jaws are the most likely drivers of this final stage of handling. But two of our findings point to underexplored, potentially fascinating questions. First, the branchial basket may be more important in food handling than we have realized. In predatory fishes, the pharyngeal jaws are often the focus of prey handling, while the importance of the other branchial arches is usually stressed in filter feeders. The apparent recruitment of the branchial basket for food handling in channel catfish suggests that its role in feeding more generally is worth further investigation. Second, and a more unexpected finding, is that peristalsis may not be the sole driver of esophageal transport in catfishes. Whether the observed correlations between cranial expansion and esophageal food transport are causative or mere coincidence remains to be seen, but the phenomenon we describe here is certainly worth further investigation.

## Supplementary Material

obaa018_Supplementary_Data
